# Ascertaining the impact of catastrophic events on dengue outbreak: The 2014 gas explosions in Kaohsiung, Taiwan

**DOI:** 10.1371/journal.pone.0177422

**Published:** 2017-05-17

**Authors:** Ying-Hen Hsieh

**Affiliations:** Department of Public Health and Center for Infectious Disease Education and Research, China Medical University, Taichung, Taiwan; University of Waterloo, CANADA

## Abstract

Infectious disease outbreaks often occur in the aftermath of catastrophic events, either natural or man-made. While natural disasters such as typhoons/hurricanes, flooding and earthquakes have been known to increase the risk of infectious disease outbreak, the impact of anthropogenic disasters is less well-understood. Kaohsiung City is located in southern Taiwan, where most dengue outbreaks had occurred in the past two decades. It is also the center of petrochemical industry in Taiwan with pipelines running underneath city streets. Multiple underground gas explosions occurred in Kaohsiung in the evening of July 31, 2014 due to chemical leaks in the pipelines. The explosions caused 32 deaths, including five firefighters and two volunteer firefighters, and injured 321 persons. Historically, dengue outbreaks in southern Taiwan occurred mostly in small numbers of around 2000 cases or less, except in 2002 with over 5000 cases. However, in the months after the gas explosions, the city reported 14528 lab-confirmed dengue cases from August to December. To investigate the possible impact, if any, of the gas explosions on this record-breaking dengue outbreak, a simple mathematical model, the Richards model, is utilized to study the temporal patterns of the spread of dengue in the districts of Kaohsiung in the proximity of the explosion sites and to pinpoint the waves of infections that had occurred in each district in the aftermath of the gas explosions. The reproduction number of each wave in each district is also computed. In the aftermath of the gas explosions, early waves occurred 4–5 days (which coincides with the minimum of human intrinsic incubation period for dengue) later in districts with multiple waves. The gas explosions likely impacted the timing of the waves, but their impact on the magnitude of the 2014 outbreak remains unclear. The modeling suggests the need for public health surveillance and preparedness in the aftermath of future disasters.

## Introduction

Dengue is a mosquito-borne viral disease transmitted by female mosquitoes mainly of the species *Aedes aegypti* and, to a much lesser extent *A*. *albopictus*, which has rapidly spread in all regions of the world in recent years [1]. Severe dengue (also known as Dengue Hemorrhagic Fever or DHF) affects most South/Southeastern Asian and Latin American countries and has become a leading cause of hospitalization and death among children in these regions [2]. Incidence of dengue has increased rapidly worldwide in the last decades. One recent study provides a credible estimation of 284 to 528 indicates 390, of which 96 million (67–136 million) manifest clinically with any severity [3]. Another recent study estimates that in 2012, approximately 2.5 billion people were at high risk of infection [4]. In 2010, nearly 2.4 million cases were reported to the WHO. Although the full global burden of the disease is still uncertain, it should be noted that the initiation of global efforts to record all dengue cases, along with recent advances in diagnostic techniques of dengue [5], partly explains the sharp increase in the number of cases reported in recent years.

**Fig 1:**
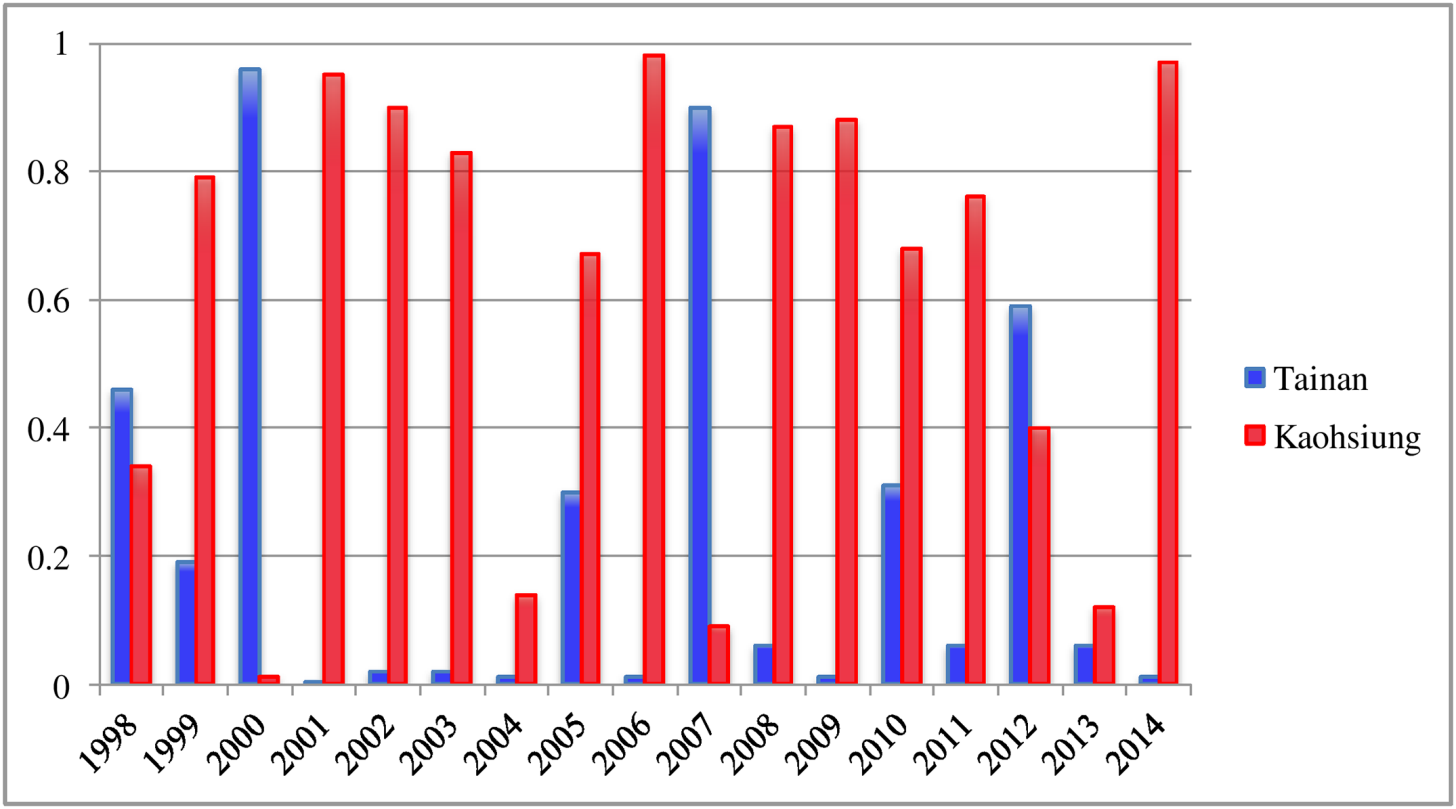
Yearly percentage of all indigenous DF/DHF cases in Taiwan that have been reported in Tainan and Kaohsiung during 1998–2014.

Taiwan is located in the tropical-subtropical region of the Northern Hemisphere, split north-south near the middle of the island by the Tropic of Cancer, with *Aedes Aegypti* manifesting mostly in the southern part. In Taiwan, there were frequent dengue outbreaks in the first half of 20th century (e.g., [6–11]). However, no outbreak had occurred in the main island of Taiwan from 1944 until 1987 [10] when martial law, first implemented in Taiwan in 1949, was revoked and traveling abroad was once again allowed. After 1987, majority of dengue cases in Taiwan were reported in the southern cities of Kaohsiung and Tainan almost every summer (Fig 1). Except for 2004 and 2013, when Tainan and Kaohsiung combined for only 15% and 18% of all indigenous cases in Taiwan, respectively, the combined percentage of indigenous cases from these two cities accounts for at least 80% of the cases every year, including some years when almost all of the cases were reported in these two cities (99% in 2005, 2006 and 2010). Moreover, for all these years of high incidence with exception of one year (1998), one of these two cities reported 59% or more of all indigenous cases that had occurred in Taiwan that particular year. The reason for this phenomenon is likely rooted in many factors including climate, molecular evolution, and geographic location.

Dengue outbreaks in Taiwan have been known to be driven initially by imported cases. Moreover, several epidemics in Taiwan during the 1980’s and 1990’s were found to be statistically significantly associated with the increasing numbers of dengue cases in several Asian countries, namely Indonesia, Thailand, Malaysia, Myanmar, the Philippines, Laos and Viet Nam, before or during the epidemic [12]. However, since 1987 dengue outbreaks in Taiwan occurred mostly in small numbers of around 2000 cases or less, except in 2002 (with over 5000 cases) and in 2014–2015 with recording-breaking DF/DHF case numbers for two consecutive years. Furthermore, the 2014 Kaohsiung outbreak was reported to be caused mainly by DENV-1 serotype [13–14].

Kaohsiung City is also the center of petrochemical industry in Taiwan with many pipelines running underneath the city streets in a complex web. Unfortunately, a series of underground gas explosions occurred in Kaohsiung in the late night of July 31, 2014 due to chemical leaks in the pipelines. The explosions caused 32 deaths, including five firefighters and two volunteer firefighters, and injured 321 persons.

Natural disasters such as typhoons/hurricanes, flooding and earthquakes have been known to increase the risk of infectious disease outbreak. In particular, flooding often leads to a proliferation of mosquitos in the disaster area [15]. The developing world has been particularly susceptible to infectious disease outbreaks in the aftermath of natural disasters [16–20], as shown for example by the report of increased dengue infections in Thailand after the 2004 Tsunami [21] and by the cholera outbreak in Haiti after the 2010 earthquake which in a little over two years caused at least 8,000 cases [22]. For a thorough discussion on the risk of post-disaster infectious disease outbreak, see Wilder-Smith [23]

The impact of anthropogenic disaster on the risk of infectious disease outbreak is less well-understood. It has been proposed that the gas explosions in Kaohsiung had likely contributed to the recording-breaking dengue outbreak that followed [13–14]. In Table 1 the yearly confirmed dengue case numbers by onset date in Kaohsiung since 2002, the year when the first major dengue outbreak occurred in the city, is provided for both before the gas explosions on 7/31 and after 8/1. For all but 3 of the previously 12 years, the proportion of cases reported after August 1 was less than that of 2014. Hence, the large proportion of cases after August 1 is uncommon, if not unprecedented, and worthy of investigation.

**Table 1:** Confirmed dengue case number by onset date before 7/31 and after 8/1 during 2002–2014. (Source: Taiwan CDC).

Year	Before 7/31		After 8/1		Total # of cases
	Case number	%	Case number	%	
2002	777	16.16%	4031	83.84%	4808
2003	37	52.11%	34	47.89%	71
2004	0	0.00%	48	100.00%	48
2005	10	7.35%	126	92.65%	136
2006	52	5.52%	890	94.48%	942
2007	62	34.25%	119	65.75%	181
2008	44	10.38%	380	89.62%	424
2009	5	0.67%	743	99.33%	748
2010	50	4.65%	1025	95.35%	1075
2011	15	1.28%	1153	98.72%	1168
2012	27	5.33%	480	94.67%	507
2013	7	10%	63	90.00%	70
2014	471	3.14%	14528	96.86%	14999

In order to investigate the possible impact of the gas explosions on the ensuing dengue outbreak in Kaohsiung City, the temporal patterns and transmissibility of the outbreak in nearby districts is analyze quantitatively with the aid of a mathematical model, the Richards model, and the 2014 daily confirmed dengue case data for each district in Kaohsiung City.

## Methods

### Data

The daily confirmed dengue case data by onset date in Taiwan from 1998 to present is available at the Taiwan Government Data Platform [24]. There are a total of 37 administrative districts in Kaohsiung. From the online dataset, the time series of daily confirmed dengue case number in 2014 for the 16 districts in the close proximity of the gas explosion sites is extracted, namely, Fengshan, Qianzhen, Lingya, Xiaogang, Xinxing, Zuoying, Yancheng, Sanmin, Linyuan, Renwu, Gushan, Qianjin, Daliao, Nanzi, Niaosong, and Dashe. Note that Qianzhen and Lingya are the two districts where the gas explosions had actually occurred.

### Mathematical model

The Richards model [25] is of the form

$$C(t)=K{\left[1+e^{-ra(t-t_{i}-(\text{ln}a)/ra)}\right]}^{-1/a},$$

where C*(t)* is the cumulative number of laboratory confirmed dengue cases on day t, with t = 0 the initial onset date at the beginning of a wave of confirmed dengue cases. *K* is the total case number of this wave, *r* is the per capita growth rate of the cumulative case number, *a* is the exponent of deviation of the cumulative case curve, and *t*_*i*_ is a turning point (or the peak) of this wave, which signifies the exact moment of an upturn or downturn in the rate of increase for the cumulative case number of this wave.

The Richards model is a phenomenological model that depicts the growth of cumulative case number but, contrary to the compartmental models, does not describe the actual disease transmission process. Three model parameters of epidemiological importance are *K*, *r*, and t_i_, which can be estimated by fitting the Richards model to the time series of cumulative case data of the outbreak, using standard software with nonlinear least-squares (NLS) approximation subroutine, e.g., SAS or MATLAB. Readers are referred to Hsieh and Chen [26] or Hsieh et al. [27] for previous applications of the Richards model to dengue.

### Reproduction number

The basic reproduction number R_0_, the average number of secondary infectious cases produced by an infectious case in a totally susceptible population in the absence of intervention measures, is given by *R*_0_ = *exp*(*rT*), where r is the per capita growth rate from the Richards model fitting and T is the serial interval, or the mean time interval from onset of one infected individual to the onset of another individual infected by him/her. It has been shown mathematically [28] that, given *r*, the expression *R*_0_ = *exp*(*rT*) provides an upper bound for basic reproduction number over any estimates obtained from all assumed distributions of the serial interval T.

## Results

**Fig 2:**
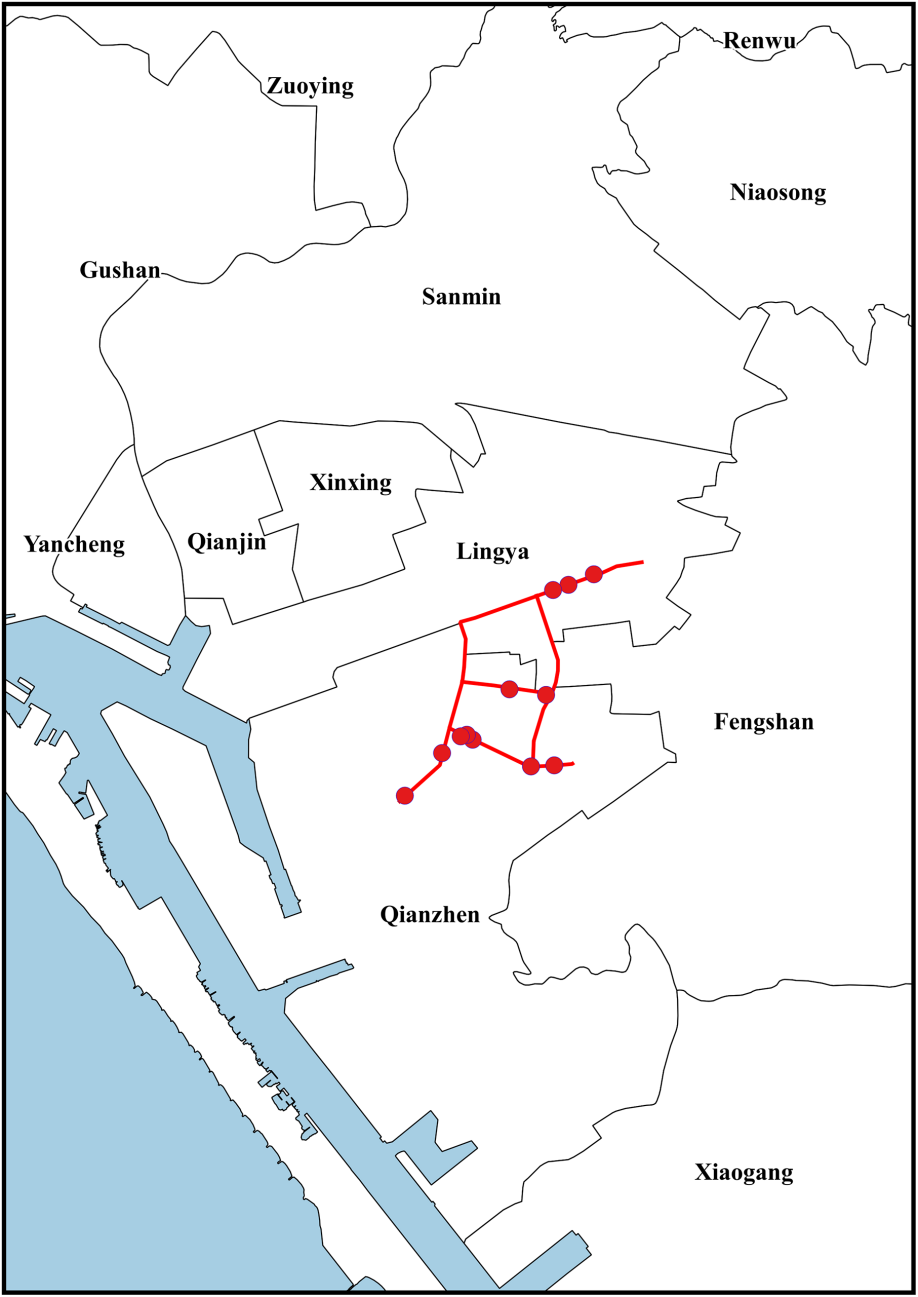
Geographic location of 2014 July 31 gas explosion sites in Kaohsiung. Red circle denotes an explosion site; red line denotes the streets that were damaged by the explosions.

**Fig 3:**
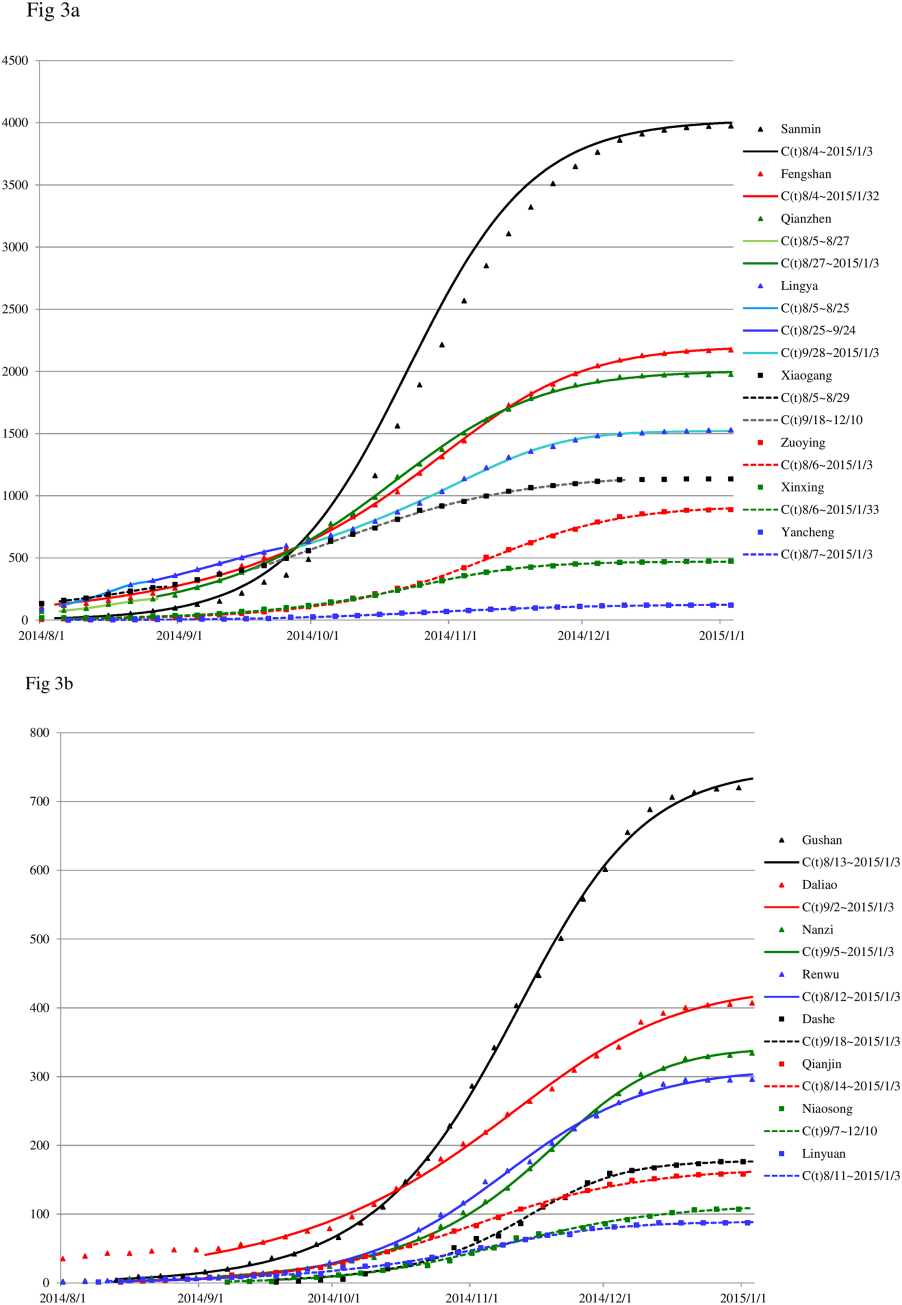
Data fit to the Richards model of lab-confirmed daily dengue cases from August 2014 to January 2015 for 16 districts near gas explosion sites and Kaohsiung City. (3a): Fengshan, Xinxing, Zuoying, Yancheng, Sanmin, Qianzhen, Lingya, Xiaogang, and Kaohsiung City; (3b): Linyuan, Renwu, Gushan, Qianjin, Daliao, Nanzi, Niaosong, and Dashe. The dots denote real data, shown every fifth day; the curves are the model fits.

The Richards model is fitted to the cumulative daily lab-confirmed dengue case numbers from August 2014 to January 2015, for each of the 16 districts in proximity of the gas explosion sites and for the whole city (see Fig 2) immediately after the gas explosion on July 31. The model fitting pinpoints waves of infection for each district as well as the total number for Kaohsiung City, as given in Table 2. The data fits for all 16 districts are given in Fig 3.

**Table 2:** Parameter estimates of waves of dengue infections for the 16 districts of Kaohsiung near the 2014 gas explosion site, and for the whole city, by fitting the Richards model.

District	Time interval	Case number K (real data)	Turning point t_i_	Basic reproduction number R_0_
Fengshan	8/4~2015/1/3	2096 (2063)	10/31	1.53~1.72
Qianzhen	8/5~8/27	102 (99)	8/19	1.96~2.55
	8/27~2015/1/3	1840 (1807)	10/20	1.75~2.05
Lingya	8/5~8/25	204 (196)	8/17	2.49~3.24
	8/25~9/24	378 (276)	9/11	1.41~1.75
	9/28~2015/1/3	916 (921)	11/3	1.31~1.41
Xiaogang	8/5~8/29	124 (121)	8/23	1.48~1.65
	9/18~12/10	756 (712)	10/6	1.63~2.00
Xinxing	8/6~2015/1/3	455 (456)	10/25	1.84~2.18
Zuoying	8/6~2015/1/3	912 (881)	11/11	1.95~2.36
Yancheng	8/7~2015/1/3	127 (120)	10/27	2.37~3.26
Sanmin	8/9~2015/1/3	4008 (3957)	10/28	2.82~3.80
Linyuan	8/11~2015/1/3	87 (85)	11/5	1.71~2.00
Renwu	8/12~2015/1/3	309 (293)	11/9	2.29~3.00
Gushan	8/13~2015/1/3	749 (721)	11/12	2.14~2.67
Qianjin	8/14~2015/1/3	167 (158)	11/2	2.10~2.68
Daliao	9/2~2015/1/3	385(359)	11/11	1.52~1.73
Nanzi	9/5~2015/1/3	334 (325)	11/21	1.89~2.25
Niaosong	9/7~2015/1/3	114 (107)	11/8	2.41~4.04
Dashe	9/18~2015/1/3	178 (177)	11/15	2.27~3.02
Kaohsiung City	8/5~8/28	904 (716)	8/20	1.80~2.29
	10/22~12/31	8287 (8000)	11/1	1.61~1.90

**Fig 4:**
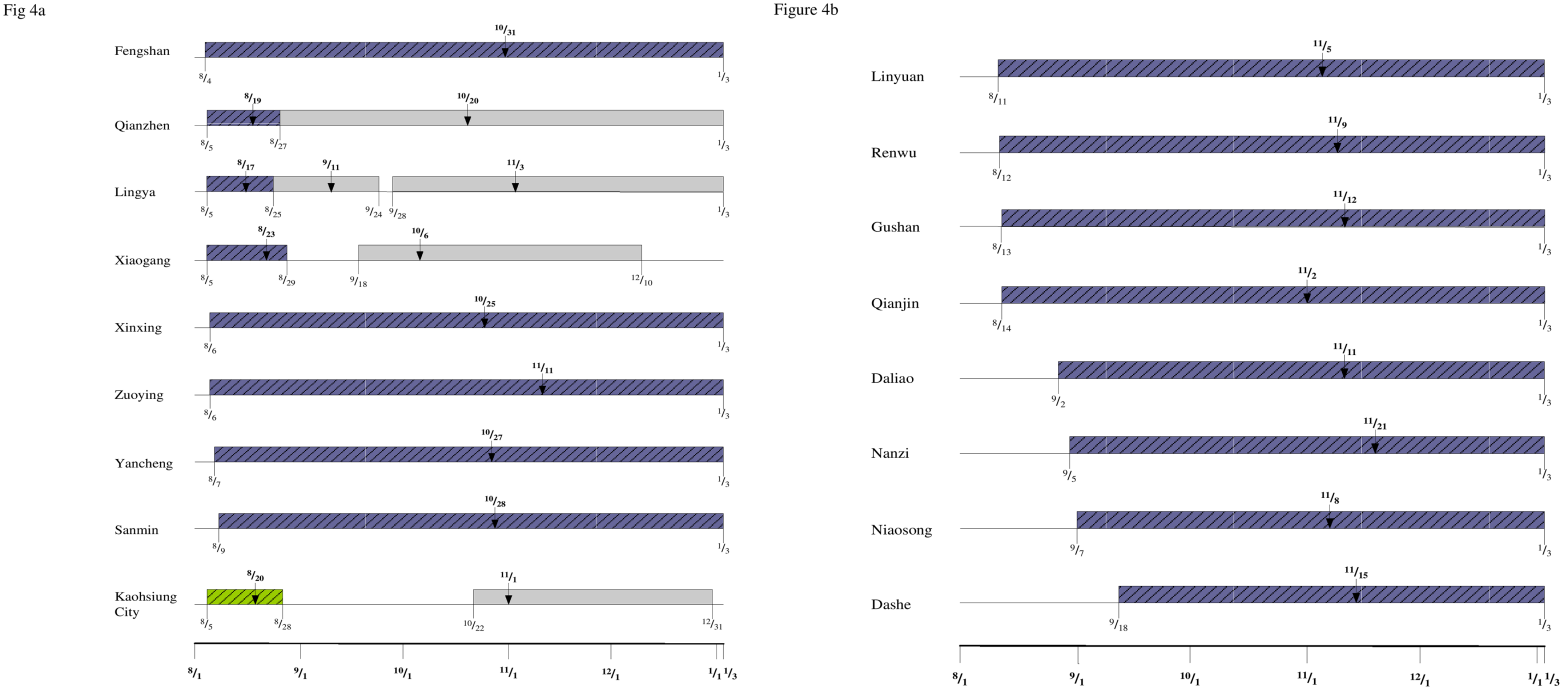
Timelines for waves of dengue infections after the gas explosion in 16 districts near the gas explosion sites. (4a): Fengshan, Xinxing, Zuoying, Yancheng, Sanmin, Qianzhen, Lingya, Xiaogang, and Kaohsing City; (4b): Linyuan, Renwu, Gushan, Qianjin, Daliao, Nanzi, Niaosong, and Dashe. The diagonally shaded blocks denote the first wave for each district; dates with arrows denote the turning point of each wave.

**Fig 5:**
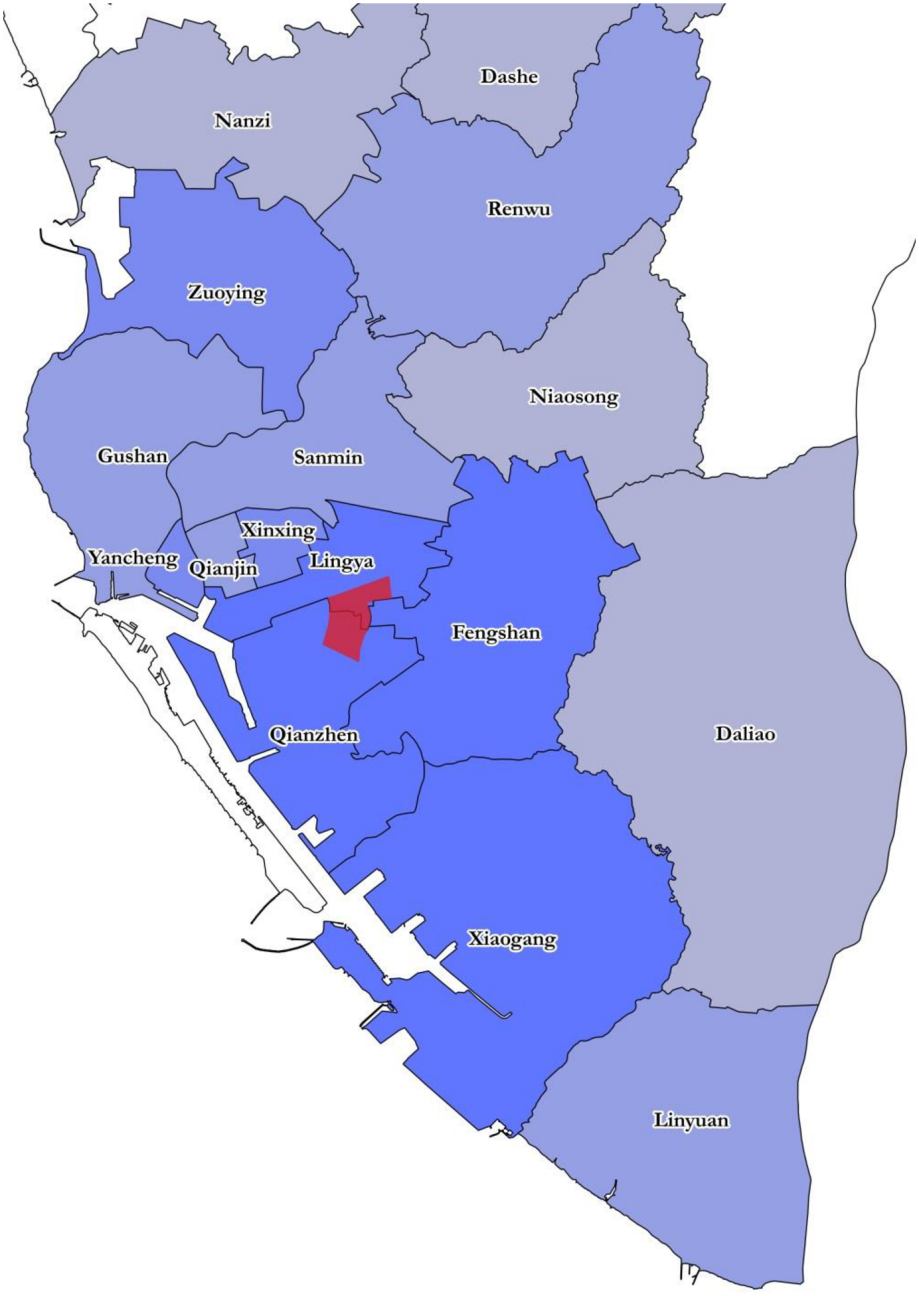
Map of spatial/temporal progression of dengue after the gas explosions in the 16 districts surrounding the gas explosion area (in red) with four levels of blue colors representing four groups of districts classified by the starting time of the waves: of August 4–5; August 6–7; August 9–14; and September 2–18. Red denotes the gas explosion area.

To further illustrate and compare the temporal progression of the waves, timelines of the waves of infections in each district are provided in Fig 4, with that of Kaohsiung City at the bottom of Fig 4a. The spatial/temporal progression of the waves is illustrated geographically with a map in Fig 5, where the districts are grouped into four temporal groups in accordance with the starting date of the first wave of infections for each district in the aftermath of the gas explosions as shown in Table 2.

**Fig 6:**
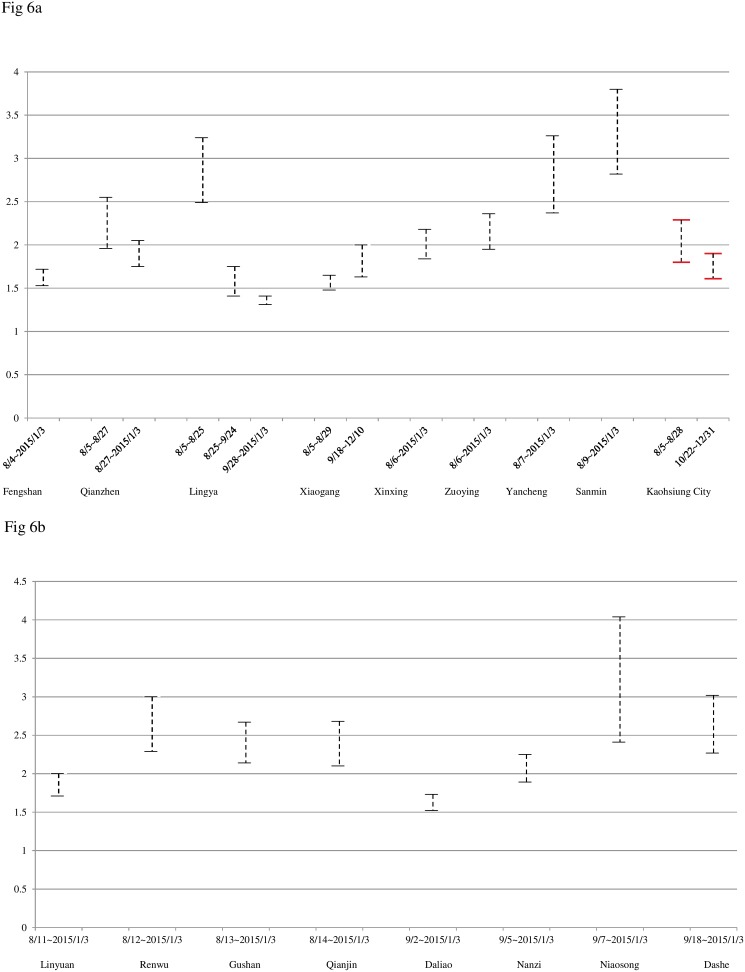
Estimated ranges of basic reproduction numbers for waves of dengue infections after the gas explosion in 16 districts near the gas explosion sites. (6a): Fengshan, Xinxing, Zuoying, Yancheng, Sanmin, Gushan, Qianjin, Kaohsiung City; (6b): Qianzhen, Lingya, Xiaogang.

To further ascertain the geographical heterogeneity in transmission potential, we also compute the estimated range of basic reproduction number *R*_0_ for each wave of infections, as given in Table 2 and illustrated in Fig 5. The estimated range was computed using the formula *R*_0_ = *exp*(*rT*). The range of *R*_0_ was computed using the 95% CI of r in Table 2 the range of T is 15 days~19 days [29–30]. The results are given illustratively in Fig 6.

## Discussion and conclusion

In the aftermath of the gas explosions on the late night of July 31, he earliest waves of dengue cases detected through the modeling had occurred between August 4–5, or 4–5 days after the gas explosions (Table 2), in four districts (Fengshan, Qianzhen, Lingya and Xiaogang) near the gas explosion sites. Two of these districts, namely, Fengshan and Qianzhen, are in fact the districts where the explosions had occurred. As the multiple explosions of underground petrochemical pipelines completely uplifted whole sections of several streets (as shown in yellow in Fig 2) in the old and densely populated areas in central Kaohsiung City, one could deduce that many mosquitos populating underground sewer, and elsewhere in the nearby area, were killed, but those that survived would fly to surrounding areas and seek blood in the following days. Therefore, there was a very likely increase in mosquito activity not only in the neighborhood of the affected streets, but also in the nearby areas.

Considering an intrinsic incubation period of 4–7 days for human dengue infection [31] and ranging between 3–14 days, symptoms for dengue fever could begin as early as three days after the mosquito bite. Therefore, it is plausible that some of the local residents bitten by infected mosquitos (as Table 3 provides evidence that dengue infections were occurring in these areas prior to the gas explosions) during the first two days after the gas explosions due to increased mosquito activity could have started to show onset of symptoms after 4–5 days, which is the beginning of a wave of cases as observed. Since the data used in this study is by onset date, the starting time of the waves of *infection*s in these districts must be at least three days before August 4–5, or August 1–2, which, interestingly, coincides with the timing of the immediate aftermath of the gas explosions. In other words, if the gas explosions were to have any impact in the outbreak, it must have occurred at least three days after the event. Since the explosions occurred during late night on July 31, it follows that the starting date of waves in the four nearby districts is August 4–5.

**Table 3:** Confirmed dengue case number by onset date in Kaohsiung City in 2014, before the gas explosions on July 31, 2014.

District	Case number	%
Qianzhen	73	15.50%
Lingya	77	16.35%
Fengshan	105	22.29%
Xiaogang	122	25.90%
All other districts	94	19.96%
Total	471	100%

Interestingly, while for most of the districts, a single wave of infections started shortly after the gas explosions and lasted through the end of the year, Xiaogang and Qianzhen, the latter being one of two districts in which the gas explosions had occurred, had two waves of cases during the same time period, similar to the model fit for the whole city. Moreover, Lingya, the other district with gas explosions, had three waves. Dengue outbreaks are known to often occur in waves, as being vulnerable to environmental changes and extreme weather events [26–27]. In this study, the subsequent waves always exhibit a decreasing trend in transmissibility, which is not always the case from previous studies of waves of dengue outbreaks (see, e.g., [27]). The cut off point for the first and second waves in all three districts occurred between August 25–29, shortly before all primary and secondary schools in Taiwan reopened for the fall on September 1 of that year. Although it is unclear if any correlation exists between dengue incidence and school activity, this might be a worthwhile subject for future investigation.

Although the starting time of the waves four to five days after the gas explosions appears to indicate that the gas explosions have had some impact on the timing of the waves that had occurred, the question remains whether it played a role in the record-breaking case number reported that year. From Table 1, clearly the dengue case number before the gas explosions in 2014 was significantly higher than that of the previous three years prior to July 31. Therefore, it is conceivable that there would have been a large outbreak in Kaohsiung City in 2014 anyway, regardless of whether the gas explosions had occurred on July 31. Moreover, Table 3 shows that more than 80% of the cases that had onset prior to the gas explosions on July 31 had occurred in Qianzhen, Lingya, Fengshan and Xiaogang, the exact four districts with the earliest waves, all starting between August 4–5 (see Table 2 and Fig 4).

The results on R_0_ also indicate geographical heterogeneity in transmissibility (Fig 6). Although the four districts near the gas explosion sites with the earliest starting date of its wave of cases did not appear to have a significantly higher R_0_, the district with most reported cases, Sanmin, does have the highest estimated range of R_0_ (2.82~3.80). However, previous modeling studies of local dengue outbreaks using the same modeling methodology (Richards model) reveal that districts having a higher transmissibility does not necessarily imply a more sizable outbreak than neighboring districts [27].

Clearly, any impact of the gas explosions, whether to temporarily reducing mosquito population in the immediate vicinity, upset mosquito activity, or disperse the mosquitos, is only directly related to mosquito population. Their impact on the spread of dengue, through the temporal and geographic distribution of reported cases, can only be indirectly observed. The timing of the waves of reported onset of cases occurring 4–5 days after the gas explosions coincides with the incubation time of human infections of dengue, and thus provides indirect evidence that the gas explosions might have had an impact on the *timing* of a wave of dengue transmissions. However, it is unclear whether it has had any impact on the magnitude of the cases in Kaohsiung City that was observed in the aftermath of this disaster. Therefore, this study does not necessarily arrive at any definite conclusion regarding the impact of these gas explosions on the risk and scope of infectious disease outbreaks. Rather, the result of modeling analysis reveals possible connection between this anthropogenic disaster and the timing of waves of dengue infections that follows, as observed through case onset data.

Finally, although it has been proposed that the gas explosion had likely led to the recording-breaking outbreak in 2014 in Kaohsiung [13–14], no catastrophic events nor unusual climatological event such as typhoon with heavy rainfall had been recorded in Taiwan in the following year of 2015. However, in August-September 2015 an even more severe dengue outbreak occurred in Tainan, a city 50 km north of Kaohsiung, followed by a similarly severe outbreak in Kaohsiung two months later in October-December. Each of these two 2015 outbreaks resulted in more reported cases than the record-breaking Kaohsiung outbreak in 2014. The total case number has exceeded 40000 by early December, which is more than the combined total of the previous 17 years, from 1998–2014.

Nonetheless, one important factor for the large number of laboratory confirmed cases being reported in 2015 was due to change in diagnostic policy during the ongoing outbreak in Tainan in September of 2015, when considerably more SN1 antigen tests were performed which allowed rapid confirmation of a large number of cases shortly after the onset of illness symptoms [32]. Other factors that might have led to these two consecutive years with record-breaking massive dengue outbreaks and, more importantly, the question of whether dengue has become endemic in Taiwan with large, yearly outbreaks, are topics for future research. The result of this modeling study suggests the need for public health surveillance and preparedness in the aftermath of future disasters.

## Supporting information

S1 Dataset Raw data used in study. (XLSX)
